# Development, Demonstration, and Evaluation of Routine Monitoring of Aerosol Carbon, Oxygen, and Sulfur Content

**DOI:** 10.1021/acsestair.3c00059

**Published:** 2024-04-29

**Authors:** Purushottam Kumar, James F. Hurley, Nathan M. Kreisberg, Braden Stump, Patricia Keady, Andrew Grieshop, Gabriel Isaacman-VanWertz

**Affiliations:** † Department of Civil and Environmental Engineering, Virginia Tech, Blacksburg, Virginia 24061, United States; ‡ Aerosol Dynamics Inc., 935 Grayson Street, Berkeley, California 94710, United States; ¶ Aerosol Devices Inc.,1613 Prospect Park Way, Ste 100, Fort Collins, Colorado 80525, United States; § Department of Civil, Construction, and Environmental Engineering, North Carolina State University, Raleigh, North Carolina 27695, United States

**Keywords:** aerosol composition, instrumentation, aerosol measurement, routine monitoring, organic carbon, atmospheric chemistry

## Abstract

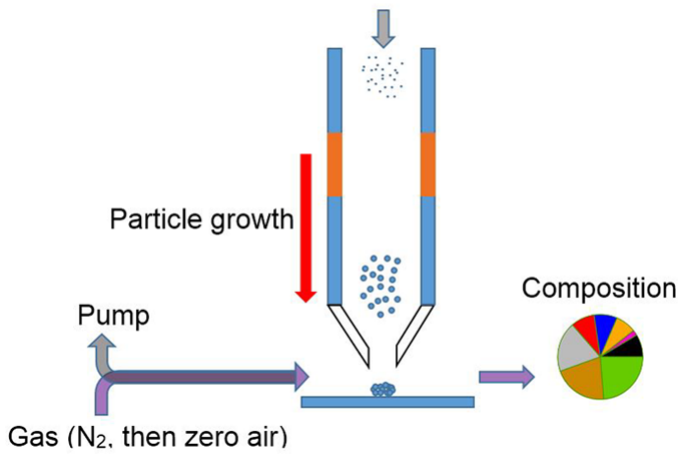

Traditional online measurements of the chemical composition of particulate matter have relied on expensive and complex research-grade instrumentation based on mass spectrometry and/or chromatography. However, routine monitoring requires lower-cost alternatives that can be operated autonomously, and such tools are lacking. Routine monitoring of particulate matter, especially organic aerosol, relies instead on offline techniques such as filter collection that require significant operator effort. To address this gap, we present here a new online instrument, the ”ChemSpot”, that provides information on organic aerosol mass loading, volatility, and degree of oxygenation, along with sulfur content. The instrument grows particles with water condensation, impacts them onto a passivated surface with low heat capacity, and uses stepped thermal desorption of analytes to a combination of flame ionization detector (FID) and flame photometric detector (FPD) and then to a CO_2_ detector downstream of the FID/FPD setup. By relying on detectors designed for gas chromatography, calibration is achieved almost entirely through the introduction of gases without the need for regular introduction of particle-phase calibrants. Particle collection efficiency of greater than 95% was achieved consistently, and the collection cell was shown to rapidly and precisely heat to ∼800 °C at a rate as fast as 10 °C per second. Measurements of total organic carbon, volatility distribution of organic aerosol, total sulfur, and oxygen-to-carbon ratio (O:C) collected during a continuous multi-week period are presented here to demonstrate the autonomous operation of ”ChemSpot”. Colocated measurements with a mass spectrometer, an aerosol chemical speciation monitor (ACSM), show good correlation and relatively low bias between the instruments (mean absolute percentage error of 21% and 27% for organic carbon and equivalent sulfate measurements, respectively).

## Abstract 2

This study demonstrates a new instrument capable of simpler, automated routine monitoring of critical aspects of the chemical composition of atmospheric aerosols to improve widespread understanding of aerosol impacts.

## Introduction

Aerosols have significant impacts on atmospheric chemistry, human health, and the Earth’s climate. Depending on their physical and chemical properties, aerosols can either scatter or absorb sunlight, affecting the Earth’s radiative balance and cloud formation. Moreover, aerosols can act as sites for chemical reactions that can alter the composition of the atmosphere^1^ and influence the ozone layer.^2^ However, routine monitoring often reports only the suspended particulate mass and does not make the chemical composition measurements necessary to fully understand the impacts of the suspended mass (e.g., source apportionment of aerosols). While U.S. monitoring networks, such as IMPROVE and CSN, collect 24 h filter samples every 3 days at nearly 300 locations, most regulatory monitoring stations around the world do not routinely collect hourly, daily, or even weekly aerosol chemical composition samples. Yet such time-resolved chemical data are key to advancing our understanding of the effects, sources, and formation mechanisms of atmospheric aerosols.^3,4^ The lack of continuous chemical composition data limits comprehension of atmospheric aerosol transformations such as growth and loss through condensation, chemical reactions, and volatilization. Improved chemical composition analysis would improve our ability to evaluate the effects of aerosols on atmospheric visibility, cloud formation and persistence, and hydrodynamic cycles. In these ways, the paucity of chemical data has hindered efforts to improve models of particulate matter and its effects on health, climate, etc.

In particular, measurements of the degree of oxygenation of aerosols (i.e., oxygen-to-carbon ratio, O:C) have been extremely limited as they generally involve mass spectrometry, which is complex and expensive. The degree of oxygenation of organic aerosols is associated with variation in their physical and chemical properties, including their ability to absorb and scatter light^5,6^ and their impact on climate and human health. Past research has shown that increases in the degree of oxygenation lead to higher hygroscopic growth of the particles,^7^ driving many of their cloud and climate impacts. Furthermore, higher polarity in organic aerosols (in other words, higher O:C) has been linked to higher toxicity.^8^ A recent study^9^ has estimated that the association between cardiorespiratory disease mortality rates in the U.S. is the highest for secondary organic aerosols (which have higher O:C than directly emitted primary organic aerosol) among different components of PM_2.5_. Due to the importance of O:C in understanding both the impacts and the chemical history of aerosols, it is widely used in reduced-parameter representations in models of organic aerosol formation and transformation in the atmosphere.^10,11^ Thus, the measurement of this parameter would considerably advance the integration of models and measurements and thereby enhance our knowledge of the complex mixture of atmospheric organic aerosols.

Currently, aerosol composition is usually measured using mass spectrometry, often in combination with different chromatographic techniques. The instruments based on these approaches provide valuable, detailed, and continuous aerosol chemical characterization, yet operational costs are significant. Calibrations often must be done manually and generally require personnel with a relatively high level of expertise. The associated costs (capital, operational/maintenance, labor) and logistical complications (external support, troubleshooting, and infrastructure) have restricted their use.

Another approach for measuring the chemical composition of aerosols involves time-integrated collection of aerosol samples on filters for gravimetric analysis or assessments using the advanced techniques mentioned above. Because sample collection on filters, extraction of chemical species, and subsequent analysis require significant operator effort, it is typically limited to time-integrated measurements every few days or weekly at a limited number of monitoring sites. Alternately, some routine monitoring programs use online thermal optical techniques to characterize the organic carbon and elemental carbon components of particles, but little information is typically reported regarding the chemical properties of the organic fraction. There are instruments for the routine measurement of aerosol mass concentration using thermal desorption to volatilize samples, with some basic composition information provided by separating organic carbon from elemental carbon.^12−15^ One complication of all thermal desorption-based instrumentation is the potential for decomposition, or specifically the potential for pyrolysis.^16^ To address this issue, many instruments use transmittance^17^ or reflectance^18^ of the sampling surface measurements to correct for any biases caused by the pyrolytic carbon. At the same time, these approaches are also susceptible to changes in the operation protocols leading to slightly different results by changing the operational conditions.^19−21^ Essentially, there is a lack of an aerosol chemical composition monitor that is cost-effective to operate, much like what is now available for gaseous constituents (e.g., monitors for O_3_, NO_X_, or SO_2_). We seek to fill this gap with an easily maintained field monitoring instrument that combines a novel, focused ultrafine particle collection, thermal transfer, and proven cost-effective gas analyzer technologies.

Presented here is an automated system, dubbed the “ChemSpot”, providing measurements of key aerosol chemical components. Measured parameters include volatility-resolved organic carbon, total aerosol sulfur, and the bulk aerosol oxygen-to-carbon ratio (O:C). The instrument is also capable of high-temperature oxidizing environments to enable measurement of elemental carbon (EC), though this parameter will not be discussed in this paper as it has not yet been validated against other established instrumentation. In this work, we demonstrate ChemSpot’s utility as an autonomous instrument for organic aerosol chemical information by examining particle collection efficiency, sample transfer, automated operation, and comparison of calibrated data to mass spectrometry-derived measurements. Measurements of ambient aerosols in Blacksburg, Virginia, not only demonstrate the validity of this instrument through colocation with other measurements but also illustrate the types of insight into aerosol properties and composition that are enabled by these data.

## Materials and Methods

The main components of the ChemSpot are (i) a condensational growth tube, (ii) a temperature cycled collection cell, (iii) a flame ionization detector (FID) and flame photometric detector (FPD) assembly, and (iv) a CO_2_ detector. Flow and heating are controlled through custom software written in LabVIEW (National Instruments). More details on the operation of FID, FPD, and CO_2_ detection can be found elsewhere.^22−25^

### Theory of Operation

The ChemSpot instrument uses condensational growth of particles to collect them onto a quartz cell surface, followed by thermal desorption and analysis using FID/FPD and CO_2_ detection. FID operation has been shown to provide complete combustion of carbon, so the total organic carbon is measured via the CO_2_ detector. The estimation of aerosol O:C ratios relies on the fact that FID response relative to the quantity of CO_2_ generated by the combustion process is a function of the degree of oxygenation of an analyte.^26^ Specifically, the FID signal produced per carbon atom (measured as the FID:CO_2_ ratio) has a negative linear correlation with O:C of the aerosol sample with an estimated error of ∼15% for complex mixtures. Sulfur content is measured using the FPD. Desorption of the sample at different temperatures provides information on volatility. The detailed operation and calibration procedure are discussed below.

### ChemSpot Prototype and Its Operation

**Figure 1:**
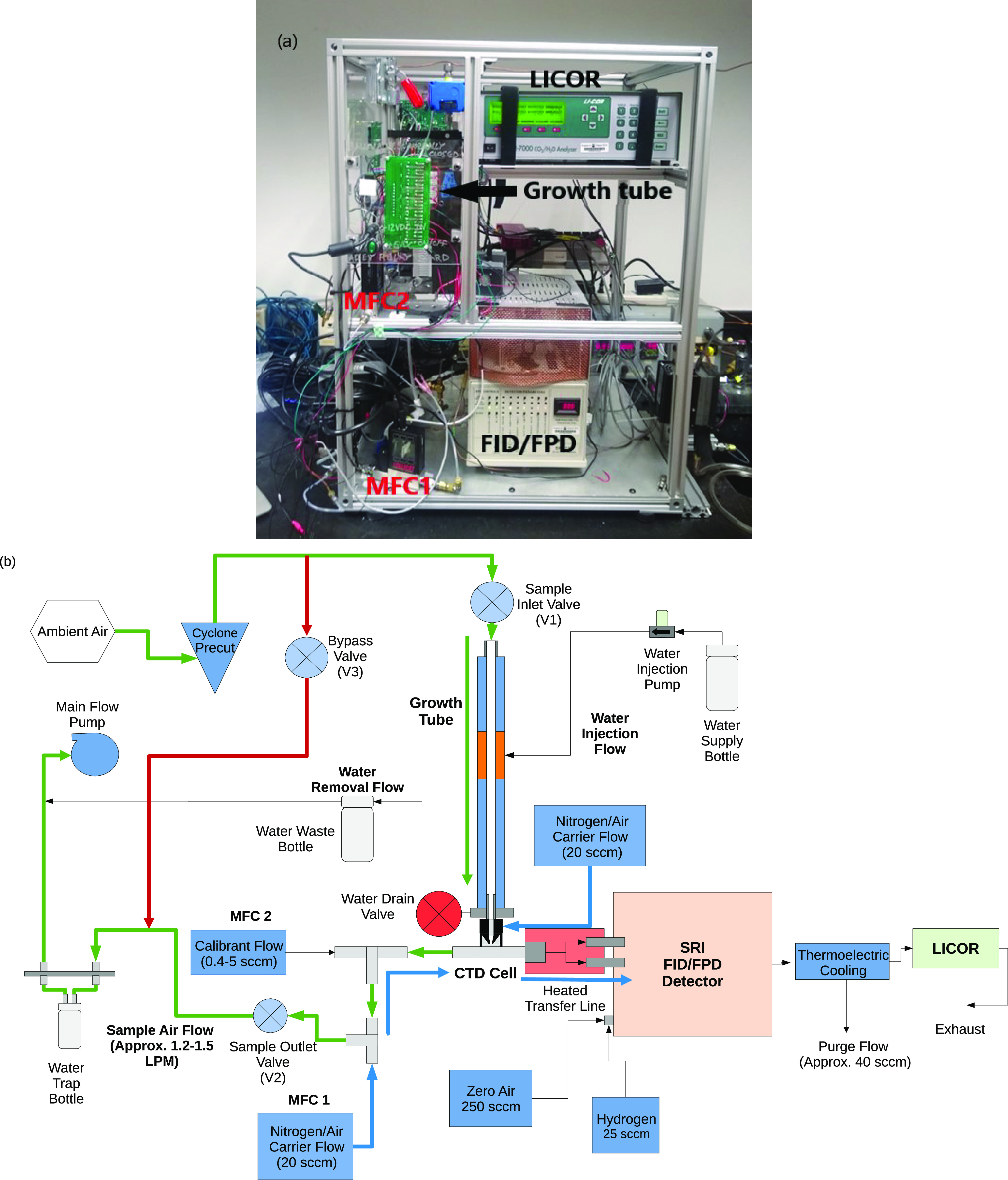
(a) ChemSpot prototype instrument (FID/FPD, LICOR, growth tube (covered in black insulation), MFC1, and MFC2 depicted in the ChemSpot image). (b) ChemSpot flow diagram. The flow path during the sampling cycle is shown in green color. Once the sampling cycle is complete, the bypass valve is opened and the flow passes through the bypass line, shown in red color. The flow path for carrier gas (and the desorbed sample) is shown in blue color.

A diagram of the ChemSpot instrument is shown in Figure 1. The instrument operates in two cycles:

*(i) Sample Collection Cycle.* Air is sampled through a PM_1_ or PM_2.5_ cyclone followed by a condensational growth tube.^27,28^ Alternating warm and cold zones generate supersaturated water vapor inside the growth tube that condenses on the fine particles as they pass through the tube, inducing particle growth. Particles grow to a size of 3–5 μm and are focused through a nozzle and impacted onto a custom-designed collection and thermal desorption (CTD) cell.^29^ The CTD cell consists of a quartz tee with a built-in impaction nozzle and is described in detail below. Condensational growth of particles has been shown to avoid significant artifacts^30−32^ and enables collection into a small ∼1 mm spot using a low-pressure differential ( ∼0.05 atm). Though condensation onto the particle might be expected to result in the uptake of gas-phase components, prior work has observed no change in composition with or without the growth tube when sampling complex organic aerosol mixtures.^30^ This is likely due to the low residence time in the growth tube (less than 100 ms) and the evaporation of the water on the surface, which may lead to the repartitioning of any absorbed vapors. Instead, the inclusion of the growth tube allows the collection of particles even down to very small sizes without the need for large pressure drops that would typically be associated with an impactor. By collecting into a very small spot onto an impaction surface, desorption temperatures are tightly controlled to minimize temperature gradients and the exposure of analytes to temperatures higher than necessary for desorption. Particle-free air is pulled out by the sample pump, passing through the sample outlet valve (V2) after the quartz cell.

*(ii) Sample Analysis Cycle.* Once the sampling cycle is complete, a bypass valve (V3) on the sampling line is opened to maintain flow through the inlet and the instrument is sealed against ambient air by closing the sample inlet valve (V1) upstream of the growth tube (which is an electronically-controlled ball valve to avoid particle loss). Inert carrier gas (nitrogen used here, controlled by a mass flow controller MFC1, MCS-100sccm, Alicat) is introduced at the downstream arm of the cell, and the coupling between the growth tube and collection cell flushes any air containing CO_2_ present in the cell out through the downstream sample outlet valve (V2) and water drain valve, which are then sealed after 2 min of purging. Closing the sample outlet valve (V2) and the water drain valve forces inert carrier gas (20 sccm flow rate) through the cell, which is heated to thermally desorb the sample through a heated transfer line (mounted in a custom aluminum manifold set at 275°C) towards the FID/FPD detector assembly (SRI Instruments, mounted on a model 110 GC chassis). The transfer line consists of a 1/32” OD capillary that also serves as the restriction between the detectors and the quartz cell, preventing the gases used for FID/FPD operation (250 sccm air and 25 sccm hydrogen) from flowing back toward the cell. The desorbed sample is combusted in the FID flame and the produced CO_2_ is passed to the CO_2_ detector. The FID temperature was maintained at 250°C throughout our experiments. The photomultiplier tube (PMT) voltage for the FPD signal was maintained at 400 V following manufacturer recommendations. Because the FID operates as a hydrogen flame, the output flow containing CO_2_ is above ambient temperature and saturated with water vapor, so flow is passed through a water removal mechanism in which a thermoelectric cooler condenses the water vapor and purges it with a minor fraction of the flow (40 sccm, or ∼20%). CO_2_ concentration in the remaining flow is measured by a commercially available CO_2_ detector (Licor 7000, LI-COR Biogeosciences). Following desorption in an inert atmosphere, the cell is heated to ∼800^°^ C in the presence of zero air. This combusts any remaining refractory carbon on the sample collection spot, which is measured as CO_2_ and provides possible quantification of any elemental carbon (EC), which will be examined in future intercomparisons. This high-temperature combustion step also minimizes interference between samples from consecutive runs by preventing the accumulation of refractory material.

The ChemSpot instrument is operated using custom-developed software in LabVIEW (National Instruments), including flow, temperature, and valve control, as well as data acquisition. The main user input panel and data acquisition/monitoring panel are shown in Figures S1 and S2, respectively. The instrument control inputs and data outputs are recorded by a custom-built control box which uses a LabJack U6-Pro for data acquisition and control. The data files generated by the software are plaintext files for easy access by the user (file format is specified by the user). The software is capable of fully automated operation of the instrument for extended periods.

### High-Temperature Passivated Collection Cell

**Figure 2:**
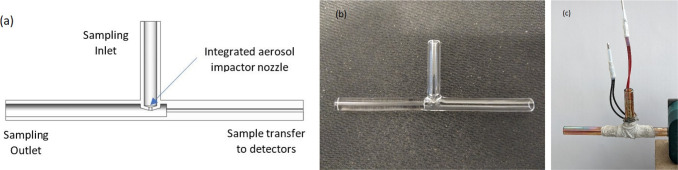
(a) CTD cell design showing the impactor nozzle and the flow directions. (b) Unpassivated CTD cell prototype without any heating wire or ceramic insulation. (c) Complete passivated cell with ceramic coating (white color section around the center of the cell) on the nickel heating wire.

These measurements rely on a new collection and thermal desorption cell (CTD) that achieves two main goals not attainable through commercially available options: (1) reaching sufficiently high temperatures (∼800^°^ C) to evolve elemental carbon, while also (2) enabling precise temperature stepping or ramping to measure thermal volatility profiles of the particle sample during thermal desorption transfer to the detectors. The cell design features a single fused quartz tee with an integrated impactor nozzle (jet diameter of 1 mm) that can collect the supermicrometer droplets (3–5 μm) generated by the coupled water condensation growth tube collector. This nozzle was integrated into the single fused assembly (Figure 2) which has an upper temperature limited only by the melting point of the material (well above 800°C). Furthermore, the low thermal conductivity of quartz allows strong temperature gradients to be maintained since it restricts heat transfer to the growth tube. This combination of features obviates the need for high-temperature seals that can greatly add thermal mass. To heat the cell quickly and precisely, the cell was wrapped with a ceramic-coated nickel wire (Figure 2c) controlled by a dedicated temperature controller to provide the heating current and measure the resulting resistance of the coil (Evolv DNA 75c controller). The temperature dependence of nickel resistivity provides a direct means of obtaining the heating coil temperature measurement without the need for an external temperature probe that would reduce temperature response. More details have been provided under the subsection “Reproducible High-Temperature Control” later in the manuscript and the Supporting Information (Figure S3). A coating of ceramic cement paste (Sauereisen Aluseal adhesive cement no. 2) was used to provide insulation to facilitate rapid heating. Passivation was applied to the cell (AMCX Inertium) to minimize the chemical activity of the surface and enhance desorption.

### Automated Gas-Phase Calibration

Calibration requires the introduction of gas-phase standards. Calibrant gas flow is controlled through a low-flow mass flow controller (MFC2 in Figure 1, MCS-10sccm, Alicat). The calibrant gas is mixed with nitrogen carrier gas upstream of the quartz cell so that the calibrant travels to detectors by the same flow path followed by ambient samples, ensuring representative calibration. Inert carrier flow controlled by MFC1 is balanced with calibrant gas flow so that total flow to detectors remains constant, avoiding changes in detector response (e.g., due to changing flame conditions). Calibrant flow rates between 0.4 and 5.0 sccm are used, providing a dynamic range of more than 10×, with <10% uncertainty in flows at all calibration levels. These low flows require only small volumes of calibrants to be used. Calibration is conducted through an automated cycle. Calibration of both CO_2_ and FID is achieved simultaneously using a hydrocarbon gas (methane, propane, butane) at cylinder concentrations on the order of 20,000 ppmC (e.g., 0.7% propane or 0.5% butane). Calibration of the FPD is achieved using SO_2_ as the calibrant gas. Gas-phase calibrations are confirmed to represent thermally desorbed samples by the introduction of liquid-phase analytes once at the start of the sampling period, with any deviations (e.g., differences in FPD response between SO_2_ and ammonium sulfate) applied as a calibration correction factor. Prior work has shown FID response to be quantitative across a wide range of organic compounds, so no such calibration factor is generally necessary for organic carbon.^26^ Instead, organic carbon measurements can be quantified using only gas-phase calibrants with occasional confirmation using condensed-phase standards (e.g., at the start of a campaign). In this work, injection of squalene at the start and end of the measurement period was used to confirm detector response. In contrast, the response of sulfur-containing compounds may vary somewhat and require a calibration factor by comparing gas-phase calibrant response to occasional introductions of ammonium sulfate solution; however, empirical investigation of this factor also suggests liquid calibration is necessary only occasionally. Calibration factors are incorporated into data collection so that the output datastream is quantitative.

### Colocated Instrumentation

For the performance evaluation of the ChemSpot instrument, an aerosol chemical speciation monitor (ACSM, Aerodyne Research Inc.) was deployed in parallel. The instrument was operated following typical manufacturer recommendations, approximately following the conditions described elsewhere (e.g., Gani et. al., 2019).^33^ The ACSM vaporizer was set at 600 °C and was equipped with a PM_1_ aerodynamic lens. The two instruments pulled Blacksburg ambient air from the same main inlet (passing through a PM_1_ cyclone, URG-2000-30E-4.4-2.5-S) and the flow was split using a Y-splitter (Brechtel 1102). Both instruments were placed within 6 ft of distance. No dryer or denuder was used on either of these two instruments.

## Results and Discussion

### Particle Collection Efficiency

**Figure 3:**
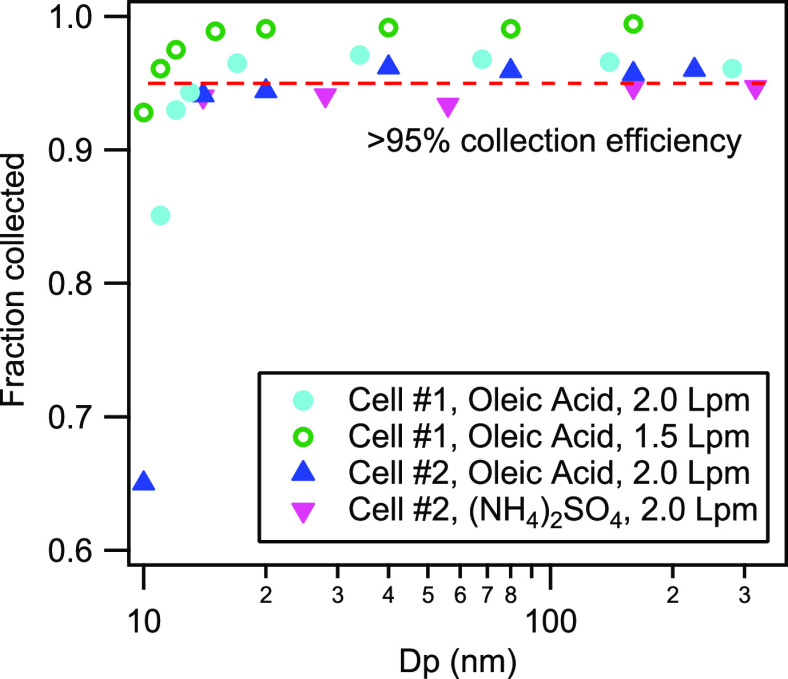
Particle collection efficiency of the CTD cell tested with two different cells at different flow rates using ammonium sulfate and oleic acid aerosols. Particle collection efficiency of >95% was achieved down to 15 nm particle size.

The quartz cell was tested for its collection efficiency as a function of particle size, composition, and flow rate. Solutions of two atmospherically relevant materials, namely ammonium sulfate and oleic acid, were atomized and sampled after passing through a differential mobility classifier to produce monomobility size fractions in the range of 10–400 nm with a 12% classification size window (8:1 sheath to aerosol flow ratio). Classified particle concentration was measured twice, immediately upstream of the growth tube and also downstream of the cell coupled to the outlet of the growth tube (i.e., particles escaping collection). For each particle size class, the ratio of these back-to-back concentration measurements equals the particle penetration fraction, *P*. The collection efficiency, *E*, equal to 1 – *P*, is shown in Figure 3 for two different cells at two flow rates (1.5 Lpm and 2.0 Lpm). Both aerosol types were collected at >95% for sizes above 15 nm with a slight improvement at the lower flow rate. Ambient aerosols were also sampled in a separate experiment without size classification for two quartz cells at 1.5 Lpm. These measurements show that the fused quartz collection cell coupled to the growth tube can collect >95% of the ambient particulate mass fraction.

### Reproducible High-Temperature Control

**Figure 4:**
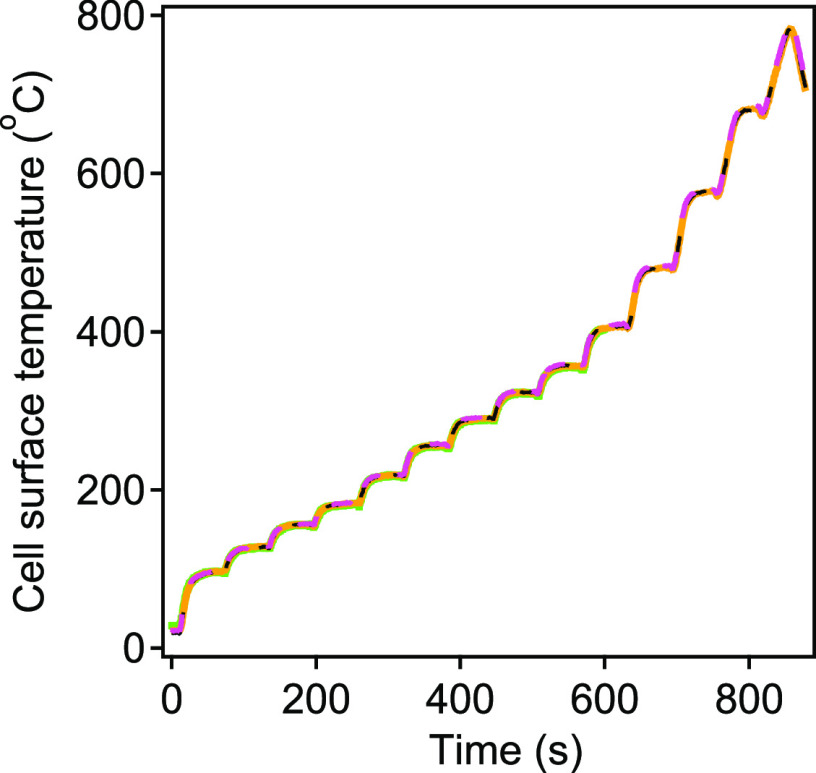
Cell desorption profile with four repeated thermal cycles (each temperature step 1 min long). Here, different colors (e.g. pink, yellow, green, etc.) represent repeated thermal cycles. A heating rate of up to 10 °C per second was achieved.

Temperature control of the quartz CTD cell was calibrated by inserting a 0.5 mm tipped thermocouple probe through the impactor nozzle to make direct contact with the interior surface of the cell where the sample is collected. The temperature of the heating element, measured directly based on the resistance of the wire, was correlated closely with the measured temperature of the internal surface of the cell. A series of calibrated responses spanning 100-800°C was used to generate a nonlinear curve that could be fit by a third-order polynomial. The fit residuals indicate better than ±4°C accuracy in capturing the reference temperature probe. More details can be found in the Supporting Information (Figure S3). Repeatability of thermal stepping is shown in Figure 4 with an overlay of measured reference surface temperatures for four repeated thermal cycles to ∼800 °C, demonstrating that the repeatability of the calibration holds over the full span of 100–800 °C. A single cell was found to withstand thousands of thermal cycles with no sign of physical or chemical instability. Though no specific investigation of the lifetime of a cell was performed, a single cell was used for more than six months, during which time it was thermally desorbed a few thousand times, providing this estimate of its minimum lifetime. The coating used to passivate the cell has been used for many years under thermal desorption environments without clear signs of decay.^34,35^ Consequently, the lifetime of each cell is expected to be long, but no quantitative lifetime could be determined.

### Autonomous Ambient Sampling and Validation

The ChemSpot instrument was run autonomously for roughly 4 weeks in June and July of 2022, sampling Blacksburg ambient air alongside an aerosol chemical speciation monitor (ACSM, Aerodyne Research Inc.) for intercomparison. The instrument ran continuously without any failures. The small missing periods in ChemSpot data were not due to instrument operating issues but rather to either human errors in specifying its operation times or reasons related to the general infrastructure or co-located instruments (power failure, sample line disturbance during the ACSM calibrations, etc.). Each data point was recorded at 3 h intervals (2.5 h for sampling and then 0.5 h for analysis) due to low-to-moderate aerosol loadings. As the system is capable of fast and precise temperature controls, in relatively higher aerosol concentration environments, hourly data points are feasible.

Particulate matter was desorbed in an inert atmosphere (nitrogen) at four successive temperature steps (100, 200, 300, and 550 °C), then desorbed in an oxidizing atmosphere (in this case, zero air) to possibly measure refractory carbon and clean the particle collection surface of residual carbon.

### Ambient Mass Distributions

**Figure 5:**
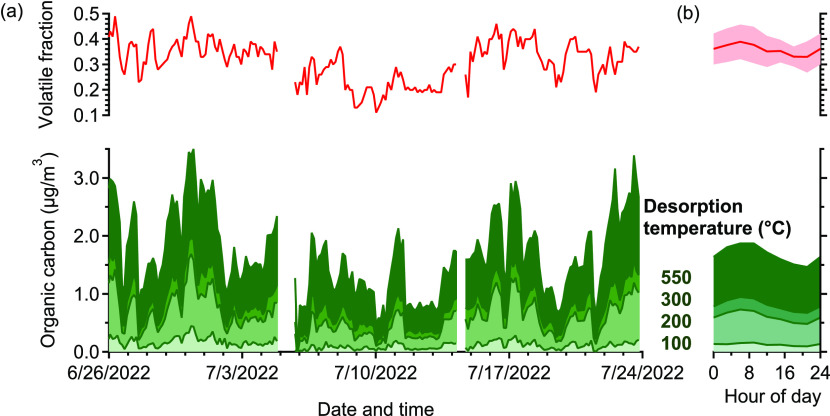
(a) Volatility separation of the measured organic carbon in four temperature bins (100, 200, 300, 550°C) and the fraction of total organic carbon measured in the first two temperature steps represented by the red line in the top graph. The distribution of organic carbon in four volatility bins varied over the course of 4 weeks of sampling period. (b) Diurnal patterns in these same parameters, volatile fraction and mass concentration of organic carbon. Diurnal data are averages within 3 h intervals, with standard deviation shown as the shaded region for volatile fraction.

In Figure 5a, the volatility distribution of measured organic carbon is shown for the 4 weeks of autonomous operation. The distribution of organic carbon across the four bins was observed to shift between samples, indicating differences in volatility. The fraction of organic carbon observed to desorb in the first two bins (at 200°C or below, depicted by the red line, written as “volatile fraction” in the figure), varies from <20% to ∼50%. Though the specific quantification of the volatility bins is not yet known, this shift between the higher- and lower-volatility bins is representative of a qualitative shift in overall volatility distribution. The aerosol mass concentrations were relatively low throughout the sample period (average organic carbon of ∼1.5 μg m^–3^). These data demonstrate the capability of this instrument to operate at relatively low organic carbon loadings, with a detection limit below 0.5 μg m^–3^ with the 3 h time resolution. As FIDs are generally known to exhibit linearity across a large dynamic range, instrument operation under high mass loadings is not expected to introduce any issues and would enable higher time resolution through lower sampling times and volumes. During these 4 weeks shown, the prototype instrument was operated autonomously with >85% uptime. While variable refractory carbon was observed, no instrument was available to compare against the measurement of refractory carbon, so this parameter will not be discussed here.

The measured diurnal variability provides insight into the trends and properties of aerosols during this period (Figure 5b). A slight morning peak is observed in total organic carbon concentrations, but this peak is driven primarily by an increase in the lower volatility fraction of organic carbon, with an increase in aerosol volatility in the morning. This may indicate that morning aerosol is more dominated by condensation of semivolatile gases (e.g., due to vehicle emissions) than due to regional influences or may be due to temperature-driven volatilization in the afternoon. Diurnal patterns in oxygen content (discussed below) provide some additional insight into these possibilities. While this short measurement period cannot fully resolve sources, these data demonstrate the utility of routine measurements of aerosol composition for understanding aerosol properties and potential sources.

### Intercomparison to Colocated Chemical Composition Measurements

**Figure 6:**
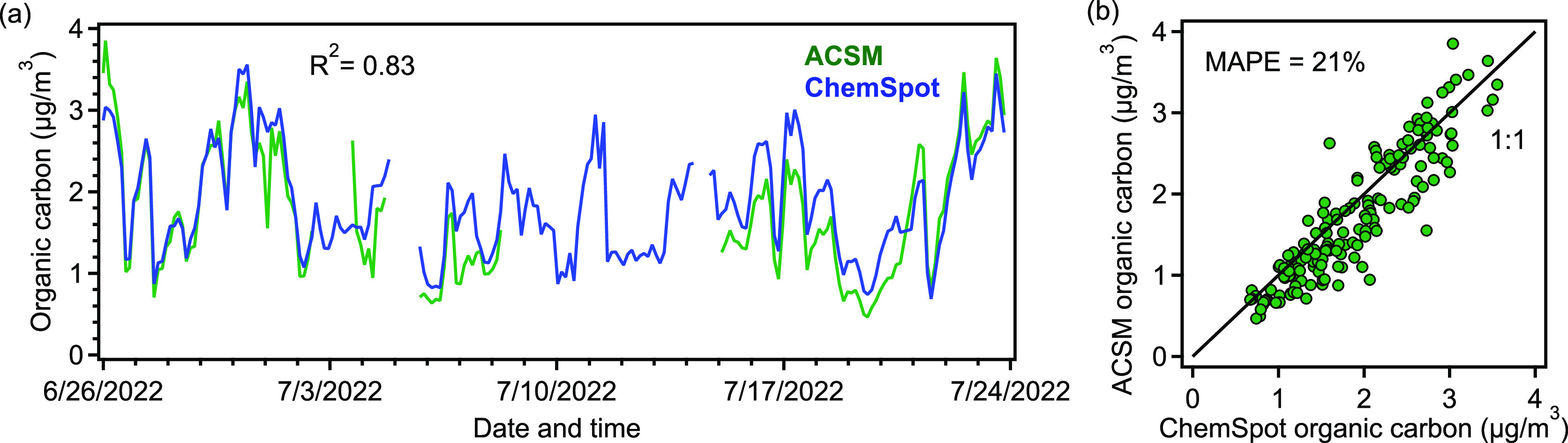
(a) Time series of aerosol organic carbon measured by ACSM and ChemSpot. (b) Scatterplot of aerosol organic carbon. The mean absolute percentage error (MAPE) of ChemSpot organic carbon measurement with respect to ACSM organic carbon measurement was 21%.

**Figure 7:**
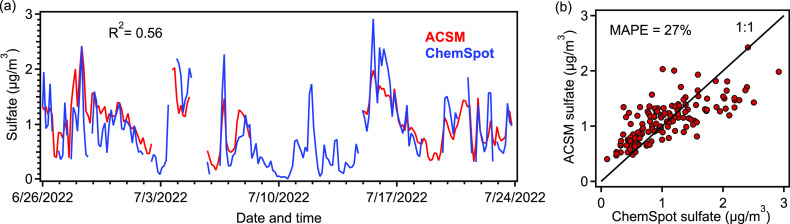
(a) Time series of sulfate measurement by ACSM and sulfur concentration (converted to equivalent sulfate mass) measured by ChemSpot. (b) Scatterplot of aerosol sulfate. The mean absolute percentage error (MAPE) of ChemSpot estimated sulfate measurement with respect to ACSM sulfate measurement was 27%.

**Figure 8:**
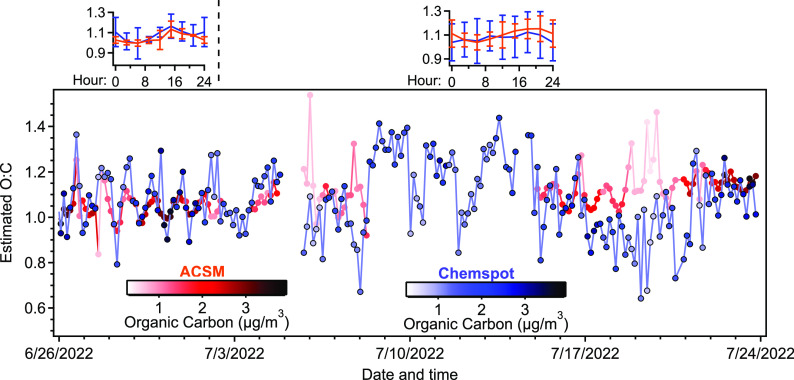
Time series of O:C over the 4 weeks of ambient sampling for the ACSM and ChemSpot depicted by orange and blue traces, respectively (shaded by organic mass concentrations). Diurnal patterns of ChemSpot are shown as insets, separated into the first week (upper left) and the last 3 weeks (upper right).

Ambient mass and chemical composition as measured by ChemSpot are shown in Figures 6–8, comparing reasonably with measurements by the ACSM. Organic carbon correlates very well with that measured by ACSM (averaged to the ChemSpot sampling periods), in terms of absolute concentrations and sample-to-sample variability (Figure 6a). Measured values from these two instruments on average differ by 21% and have an *R*^2^ of 0.83. Though the two instruments diverge slightly at times in measuring total organic carbon, the overall bias between these instruments is low (Figure 6b). During a one-week period in the middle of the deployment, the ACSM was taken offline for maintenance and ChemSpot sampled a mixture of indoor and outdoor particles, so some shifts in the chemical properties of the particles during this period were observed. The presence of other heteroatoms (nitrogen, sulfur, and phosphorus) besides oxygen in the sample has been found to have negligible effects on the FID response in our experiments. Further, the relative contribution of heteroatoms on FID response has been shown to have become less significant with increasing molecular weight.^36^

A similar comparison is observed for total sulfur, with ChemSpot-measured sulfur (converted to sulfate mass terms) consistent with the values of ACSM-measured sulfate (Figure 7). Correlation is not as good for this measurement (*R*^2^ = 0.56), and there is a slightly higher disagreement between the instruments (27%). This may be caused by calibration uncertainties from either instrument or may be due to differences in the sulfur species present, as organic and inorganic sulfur may have differing response factors that are not well constrained. Nevertheless, there is a good qualitative agreement between these instruments, and they are in general agreement throughout the operating period.

A unique capability of the ChemSpot is the online measurement of O:C without the need for an expensive mass spectrometer. The time series of O:C estimated by ChemSpot measurements during this deployment in Figure 8 is shown to correlate with that estimated using mass spectral information from the ACSM with an average O:C of roughly 1.0; the ACSM estimate is based on the fraction of observed mass spectrum that is mass spectral ion *m*/*z* 44.^37,38^ Though there is some divergence between the measurements, particularly at low particle concentrations, there are also periods of very close agreement. For example, a diurnal pattern in O:C in the first week is observed by both instruments, shown as an inset for the first week of data for ChemSpot. No clear diurnal pattern is observed for the latter period, which is true whether or not the period in the middle lacking ACSM data is included, which coincides with a period of indoor measurements instead of ambient air. Unfortunately, due to the relatively low dynamic range of O:C during this deployment, a larger range of intercomparison was not available and no scatterplot is shown.

The diurnal patterns in O:C again demonstrate some of the utility of these measurements. In the first week, a diurnal pattern is observed in which mornings have a slightly lower degree of oxygenation. During this period, the diurnal pattern in volatile fraction (Figure 5b) is actually somewhat stronger, providing possible evidence that during this period there is a stronger influence of condensation of less-polar emissions onto existing particles (e.g., influence of hydrocarbon vehicle emissions). Later in the measurement period, no diurnal pattern in O:C is observed, and the pattern in volatile fraction is weaker, suggesting a possible shift toward more regional aerosol. Again, this short period of measurements is not fully able to resolve these sources but demonstrates the value of these routine measurements of particle composition.

While the ChemSpot operation is based on the thermal desorption technique, it does not include any transmittance or reflectance-based measurement to identify and correct for pyrolytic carbon. The current design of ChemSpot focuses more on minimizing the decomposition of organic carbon by using an impactor-based sample collection approach and a cleverly optimized thermal desorption program. Still, the results of this intercomparison demonstrate that the ChemSpot instrument provides reasonable quantitative measurements of several particle chemistry parameters. Measurements of O:C and aerosol sulfur are currently not available from any other commercially available instrument without mass spectrometry. The ChemSpot enables routine monitoring of these parameters, alongside online measurements of organic carbon mass and volatility. The calibration and analysis approaches of this instrument offer several advantages for routine measurements: reliance primarily on gas-phase calibrants, simple autonomous operation, a simple plaintext data stream, and reliance on robust stable detectors.

### Atmospheric Implications

Measurements of aerosol oxygen, organic carbon, and sulfur content collected by the thermal desorption-based ChemSpot instrument are shown in this work to be in reasonably good agreement with available instruments that rely on more complex mass spectrometric methods. The present instrument quantitatively collects particles into an inert cell, with subsequent sample desorption through tightly controlled temperature profiles up to temperatures high enough to remove refractory matter. By relying on robust detectors designed for gas chromatography, detector responses to condensed phase analytes can be well described using gas-phase calibrants, facilitating simpler deployment. However, thermal instrumentation can suffer biases or uncertainty due to thermal decomposition or pyrolysis of more thermally labile particle-phase components. Nevertheless, the concentrations of organic carbon measured by this approach, including the use of gas-phase calibrants to quantify instrument response, were found to be in good agreement with the bulk mass spectrometric measurements. Effects of decomposition may not be observed in the comparison to the ACSM due to the tight thermal control of the sample achieved by impaction in a very small deposit onto a solid directly heated surface, but further investigation is warranted to better understand the impacts of pyrolysis or other decomposition processes on these measurements.

The prototype instrument was operated for a month with very little downtime and compared well to established instrumentation. The development of this system could give researchers and government agencies the capability to routinely and more easily monitor the chemical composition of aerosols (including bulk O:C of aerosols) in the comprehensive manner needed for improving current models and policies designed to curb air pollution effects. Future work remains to evaluate the instrument performance in wider ranges of aerosol loadings and source contributions, but the measurements collected here demonstrate that even relatively simple chemical composition measurements can begin to bring insight into the properties, sources, and transformations of ambient aerosols. Validation of some of the measurement parameters, such as volatility and elemental carbon, will require continued in-depth investigation as they are complex parameters that can vary depending on the method by which they are measured.^39,40^ However, overall the results of this research show great promise for the ChemSpot as a monitoring instrument for unattended measurements of particle mass and composition.

## Supporting Information Available

Screenshots of the control panel software and a figure showing CTD cell calibration (PDF)
