# Supplementary material for: Development, Demonstration, and Evaluation of Routine Monitoring of Aerosol Carbon, Oxygen, and Sulfur Content
Source: ACS EST Air. 2024 Apr 29;1(6):464–73. doi: 10.1021/acsestair.3c00059 (PMC11184560; doi:10.1021/acsestair.3c00059)
Supplement: Supplementary file 1 — ea3c00059_si_001.pdf [file ea3c00059_si_001.pdf]

Supporting information: Development, demonstration, and evaluation of routine monitoring of aerosol carbon, oxygen, and sulfur content.

Purushottam Kumar,[†] James F. Hurley,[†] Nathan M. Kreisberg,[‡] Braden Stump,[¶] Patricia Keady,[¶] Andrew Grieshop,[§] and Gabriel Isaacman-VanWertz[*,†]

†*Department of Civil and Environmental Engineering, Virginia Tech, Blacksburg, VA-24061, United States*

‡*Aerosol Dynamics Inc., 935 Grayson Street, Berkeley, CA-94710, United States*

¶*Aerosol Devices Inc.,1613 Prospect Park Way Ste 100, Fort Collins, CO-80525, United States*

§*Department of Civil, Construction, and Environmental Engineering, North Carolina State University, Raleigh, NC-27695, United States*

E-mail: ivw@vt.edu

Phone: +1 (540)-231-0011. Fax: +1 (540)-231-7916

Figure S1 represents the main DAQ setup where inputs are specified.

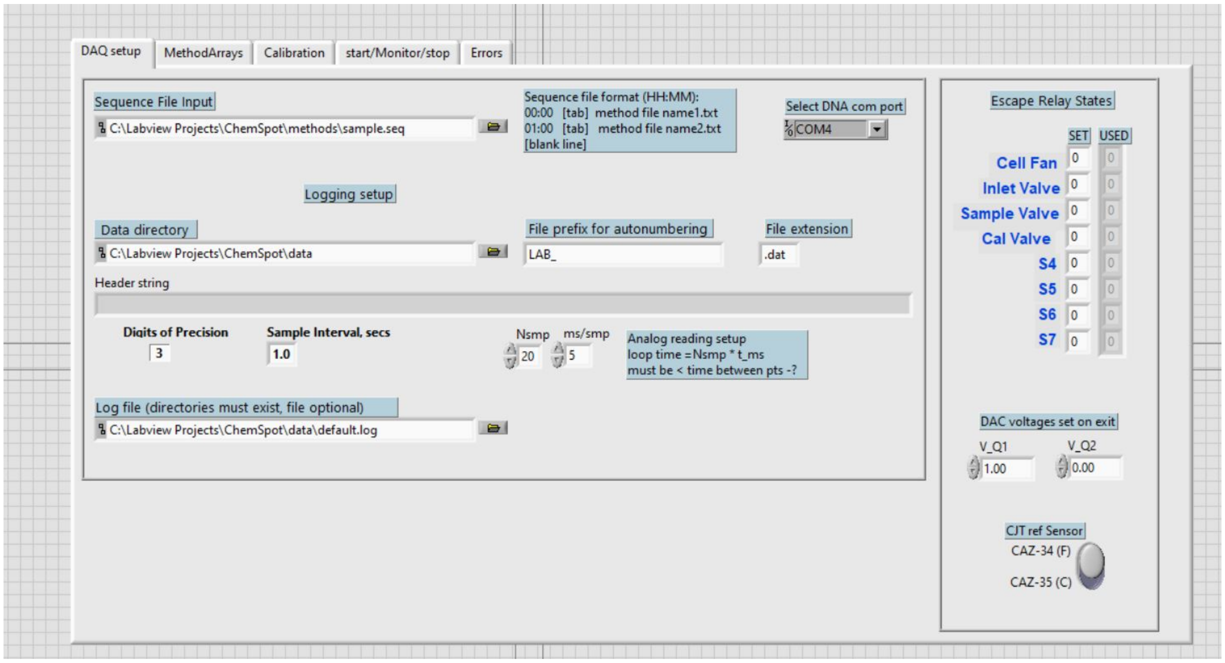

Figure S1: Main input panel

Figure S2 represents the monitoring panel where live FID, FPD, CO_2, and other parameters are shown in real-time.

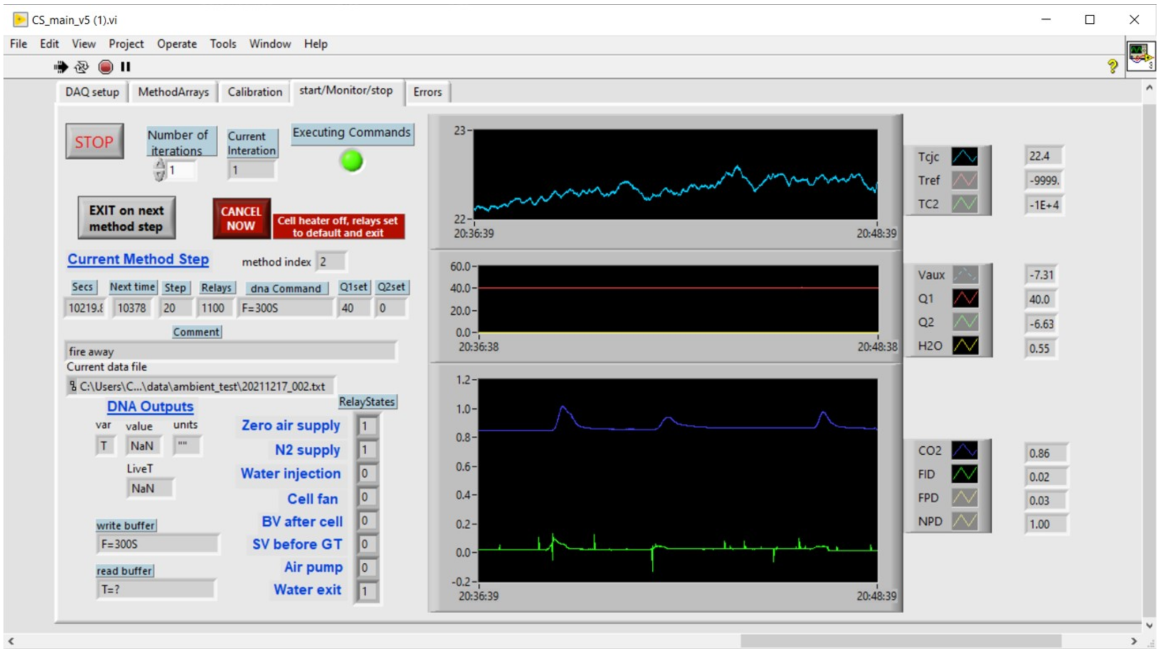

Figure S2: Monitoring panel

Figure S3 represents the calibration of CTD cell surface temperature using controller set points. Here, the y-axis, written as the controller set point is representative of the resistance set points of the heating wire and the corresponding cell surface temperature measured by an external thermocouple is on the x-axis.

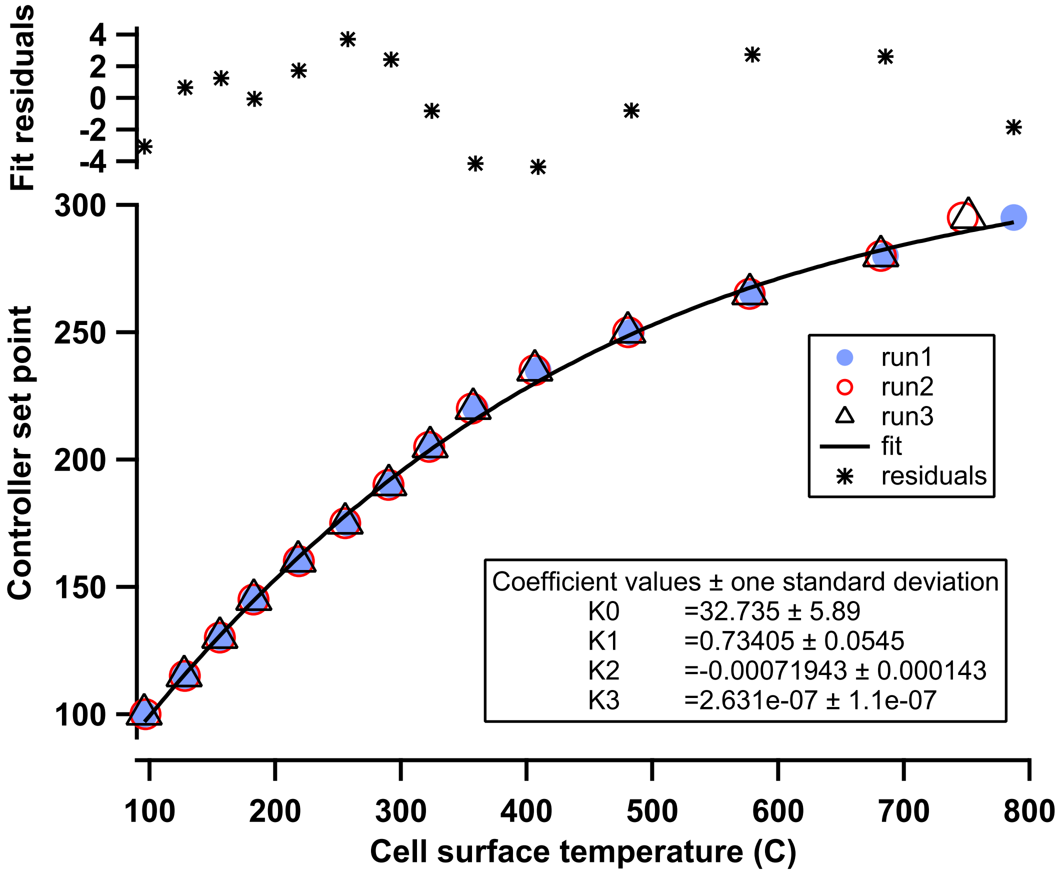

Figure S3: Cell heater calibration profile
